# 1,4-Di­methyl­piperazine-2,3-dione

**DOI:** 10.1107/S2414314624009362

**Published:** 2024-10-04

**Authors:** Themmila Khamrang, C. Ponraj, Madhukar Hemamalini, G. Jerald Maria Antony, Dhandayutham Saravanan

**Affiliations:** a Department of Chemistry Dhanamanjuri University, Manipur 795 001 India; b Department of Chemistry, National College, Tiruchirappalli, Tamil Nadu, India; c Department of Chemistry Mother Teresa Women’s University, Kodaikanal Tamil Nadu India; University of Aberdeen, United Kingdom

**Keywords:** crystal Structure, half chair, hydrogen bonding

## Abstract

In the title compound, C_6_H_10_N_2_O_2_, the piperazine-2,3-dione ring adopts a half-chair conformation. In the crystal, the mol­ecules are linked by weak C—H⋯O hydrogen bonds, forming (010) sheets.

## Graphical abstract

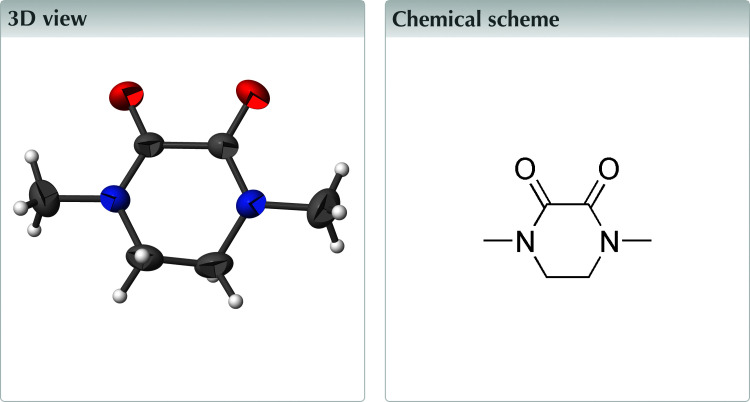

## Structure description

Piperazine and its derivatives are found within biologically active mol­ecules across a diverse range of therapeutic areas, including anti­fungal, anti­bacterial, anti­malarial, anti­psychotic, anti­depressant, and anti­tumor applications targeting colon, prostate, breast, lung, and leukemia cancers (Brockunier *et al.*, 2004 ▸; Bogatcheva *et al.*, 2005 ▸). As part of our studies in this area, we now describe the structure of the title compound, C_6_H_10_N_2_O_2_.

**Figure 1:**
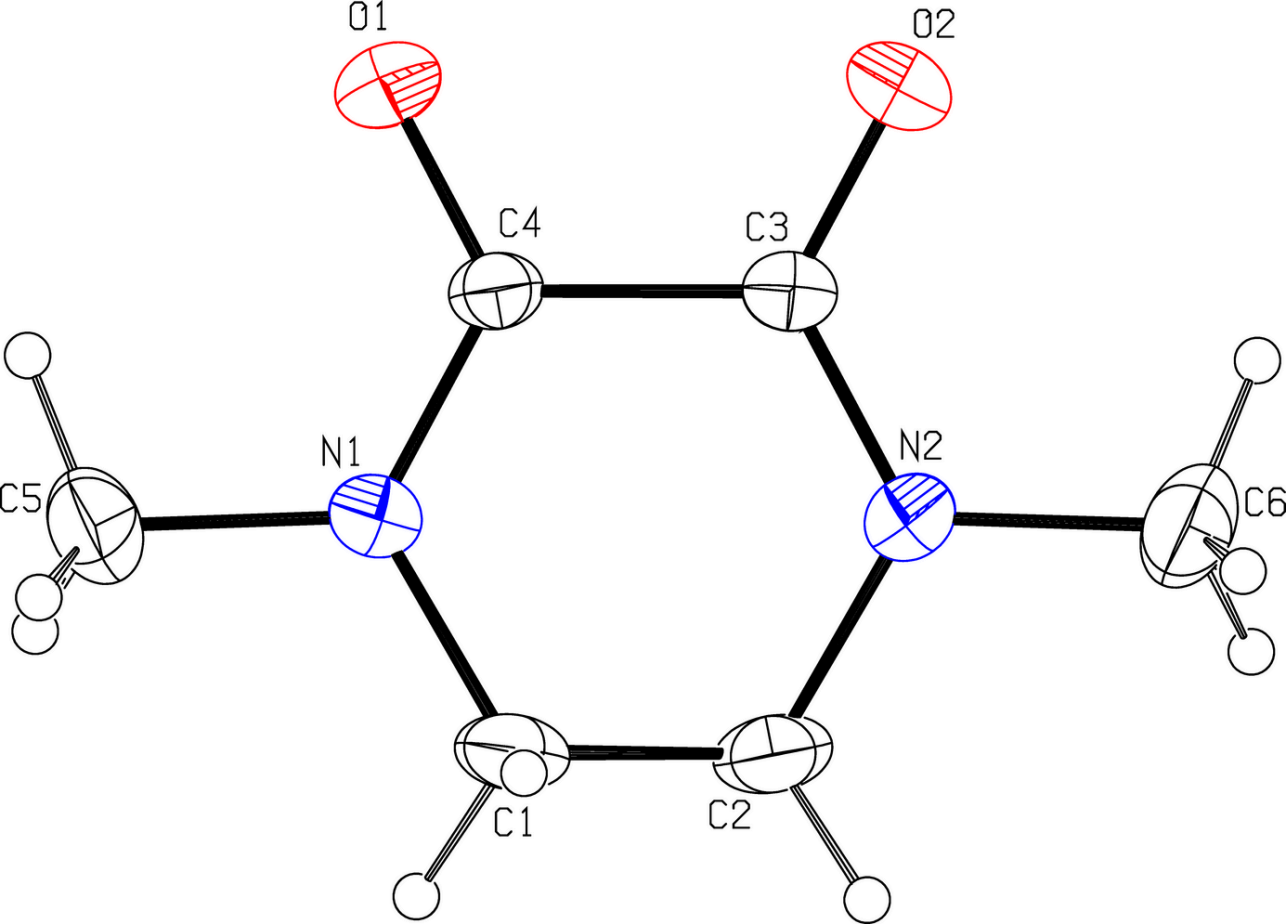
The asymmetric unit with displacement ellipsoids drawn at the 50% probability level.

**Figure 2:**
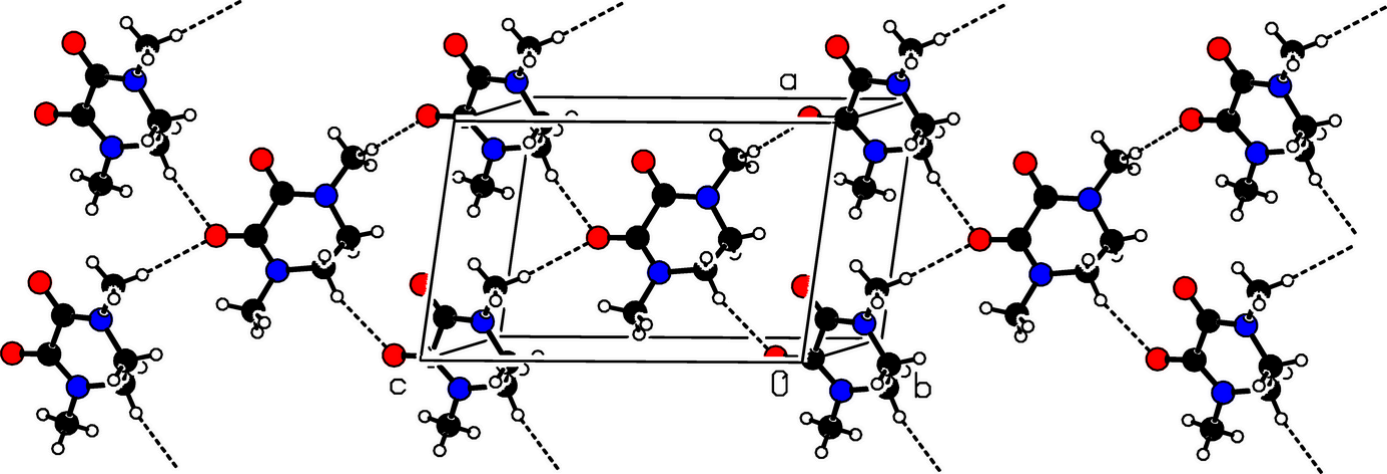
The crystal packing of the title compound.

The asymmetric unit is shown in Fig. 1 ▸. The piperazine-2,3-dione ring adopts a half chair conformation, with C1 and C2 displaced from the other ring atoms by 0.279 (3) and −0.342 (3) Å, respectively. The mol­ecule possesses local *C*_2_ symmetry about an axis passing through the midpoints of the C1—C2 and C3—C4 bonds. In the crystal (Fig. 2 ▸), the mol­ecules are connected by weak C2—H2*A*⋯O1 and C5—H5*C*⋯O2 hydrogen bonds (Table 1 ▸) to generate (010) layers.

A search of the Cambridge Structural Database (CSD; Version 5.43, update November 2022; Groom *et al.*, 2016 ▸) revealed some similar structures to the title compound, including 3,6-di­benzyl­idene-1,4-di­methyl­piperazine-2,5-dione (CSD refcode IQOCEZ; Ge *et al.*, 2019 ▸), 2,5-bis­(1-methyl-2-oxoindol-3-yl­idene)-1,4-di­methyl­piperazine-3,6-dione acetone solvate (PALVUT; Gompper *et al.*, 1992 ▸) and 6-(bromo­benz­yl)-3-benzyl­idene-6-*erythro*-hy­droxy-1,4-di­methyl­piperazine-2,5-dione (SAWSEO; Sterns *et al.*, 1989 ▸).

## Synthesis and crystallization

The title compound was prepared according to the literature method (Haraguchi *et al.*, 2015 ▸). Recrystallization of the solid from di­chloro­methane solution gave colorless plates, which were suitable for X-ray diffraction.

## Refinement

Crystal data, data collection and structure refinement details are summarized in Table 2 ▸.

## Supplementary Material

Crystal structure: contains datablock(s) global, I. DOI: 10.1107/S2414314624009362/hb4484sup1.cif

Structure factors: contains datablock(s) I. DOI: 10.1107/S2414314624009362/hb4484Isup2.hkl

Supporting information file. DOI: 10.1107/S2414314624009362/hb4484Isup3.cml

CCDC reference: 2386002

Additional supporting information:  crystallographic information; 3D view; checkCIF report

## Figures and Tables

**Table 1:** Hydrogen-bond geometry (Å, °)

*D*—H⋯*A*	*D*—H	H⋯*A*	*D*⋯*A*	*D*—H⋯*A*
C2—H2*A*⋯O2^i^	0.97	2.49	3.419 (3)	161
C5—H5*C*⋯O2^ii^	0.96	2.54	3.481 (3)	168

Symmetry codes: (i) 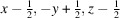; (ii) 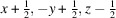.

**Table 2:** Experimental details

Crystal data	
Chemical formula	C_6_H_10_N_2_O_2_
*M*_r_	142.16
Crystal system, space group	Monoclinic, *P*2_1_/*n*
Temperature (K)	293
*a*, *b*, *c* (Å)	7.3781 (6), 8.0050 (6), 12.1306 (8)
β (°)	99.767 (7)
*V* (Å^3^)	706.07 (9)
*Z*	4
Radiation type	Mo *K*α
μ (mm^−1^)	0.10
Crystal size (mm)	0.37 × 0.32 × 0.29

Data collection	
Diffractometer	Agilent Xcalibur, Atlas, Gemini
Absorption correction	Analytical (*SADABS*; Krause *et al.*, 2015 ▸)
*T*_min_, *T*_max_	0.507, 0.578
No. of measured, independent and observed [*I* > 2σ(*I*)] reflections	2746, 1624, 1194
*R*_int_	0.016
(sin θ/λ)_max_ (Å^−1^)	0.681

Refinement	
*R*[*F*^2^ > 2σ(*F*^2^)], *wR*(*F*^2^), *S*	0.063, 0.181, 1.07
No. of reflections	1624
No. of parameters	93
H-atom treatment	H-atom parameters constrained
Δρ_max_, Δρ_min_ (e Å^−3^)	0.45, −0.21

Computer programs: *CrysAlis PRO* (Agilent, 2012 ▸), *SHELXT2018/2* (Sheldrick, 2015*a* ▸), *SHELXL2018/3* (Sheldrick, 2015*b* ▸) and *PLATON* (Spek, 2020 ▸).

## full crystallographic data

### 1,4-Dimethylpiperazine-2,3-dione Crystal data

**Table:**

C_6_H_10_N_2_O_2_	*F*(000) = 304
*M**_r_* = 142.16	*D*_x_ = 1.337 Mg m^−^^3^
Monoclinic, *P*2_1_/*n*	Mo *K*α radiation, λ = 0.71073 Å
*a* = 7.3781 (6) Å	Cell parameters from 9307 reflections
*b* = 8.0050 (6) Å	θ = 3.5–26.4°
*c* = 12.1306 (8) Å	µ = 0.10 mm^−^^1^
β = 99.767 (7)°	*T* = 293 K
*V* = 706.07 (9) Å^3^	Plate, colourless
*Z* = 4	0.37 × 0.32 × 0.29 mm

### 1,4-Dimethylpiperazine-2,3-dione Data collection

**Table:**

Agilent Xcalibur, Atlas, Gemini diffractometer	1194 reflections with *I* > 2σ(*I*)
Radiation source: fine-focus sealed tube	*R*_int_ = 0.016
ω scans	θ_max_ = 29.0°, θ_min_ = 3.1°
Absorption correction: analytical (SADABS; Krause *et al.*, 2015)	*h* = −10→8
*T*_min_ = 0.507, *T*_max_ = 0.578	*k* = −10→5
2746 measured reflections	*l* = −6→16
1624 independent reflections	

### 1,4-Dimethylpiperazine-2,3-dione Refinement

**Table:**

Refinement on *F*^2^	Primary atom site location: dual
Least-squares matrix: full	Hydrogen site location: inferred from neighbouring sites
*R*[*F*^2^ > 2σ(*F*^2^)] = 0.063	H-atom parameters constrained
*wR*(*F*^2^) = 0.181	*w* = 1/[σ^2^(*F*_o_^2^) + (0.0839*P*)^2^ + 0.2479*P*] where *P* = (*F*_o_^2^ + 2*F*_c_^2^)/3
*S* = 1.07	(Δ/σ)_max_ < 0.001
1624 reflections	Δρ_max_ = 0.45 e Å^−^^3^
93 parameters	Δρ_min_ = −0.21 e Å^−^^3^
0 restraints	

### 1,4-Dimethylpiperazine-2,3-dione Special details

**Table:**

Geometry. All esds (except the esd in the dihedral angle between two l.s. planes) are estimated using the full covariance matrix. The cell esds are taken into account individually in the estimation of esds in distances, angles and torsion angles; correlations between esds in cell parameters are only used when they are defined by crystal symmetry. An approximate (isotropic) treatment of cell esds is used for estimating esds involving l.s. planes.
Refinement. All the H atoms were positioned geometrically (C—H = 0.96–0.97 Å) and were refined using a riding model, with *U*_iso_(H) = 1.2*U*_eq_(C) or 1.5*U*_eq_(methyl C).

### 1,4-Dimethylpiperazine-2,3-dione Fractional atomic coordinates and isotropic or equivalent isotropic displacement parameters (Å^2^)

**Table:**

	*x*	*y*	*z*	*U*_iso_*/*U*_eq_	
O2	0.4932 (2)	0.2262 (2)	0.62587 (12)	0.0591 (5)	
O1	0.8009 (2)	0.3382 (3)	0.55770 (14)	0.0642 (6)	
N1	0.6498 (2)	0.3592 (2)	0.38031 (14)	0.0427 (5)	
N2	0.3516 (2)	0.1982 (2)	0.44717 (14)	0.0421 (5)	
C4	0.6624 (3)	0.3190 (3)	0.48783 (16)	0.0380 (5)	
C3	0.4923 (3)	0.2422 (2)	0.52590 (15)	0.0364 (5)	
C5	0.7995 (4)	0.4489 (4)	0.3415 (2)	0.0616 (7)	
H5A	0.899882	0.462273	0.402473	0.092*	
H5B	0.756751	0.556833	0.313902	0.092*	
H5C	0.840112	0.386644	0.282610	0.092*	
C1	0.4771 (4)	0.3452 (3)	0.30300 (18)	0.0547 (7)	
H1A	0.502520	0.333921	0.227477	0.066*	
H1B	0.406408	0.446718	0.306177	0.066*	
C2	0.3670 (4)	0.2011 (3)	0.32859 (19)	0.0555 (6)	
H2A	0.245130	0.207406	0.283752	0.067*	
H2B	0.424361	0.098582	0.309359	0.067*	
C6	0.1915 (3)	0.1170 (4)	0.4791 (3)	0.0649 (8)	
H6A	0.223240	0.073267	0.553547	0.097*	
H6B	0.151882	0.027484	0.428037	0.097*	
H6C	0.093814	0.196898	0.476737	0.097*	

### 1,4-Dimethylpiperazine-2,3-dione Atomic displacement parameters (Å^2^)

**Table:**

	*U*^11^	*U*^22^	*U*^33^	*U*^12^	*U*^13^	*U*^23^
O2	0.0637 (11)	0.0816 (12)	0.0319 (8)	−0.0121 (9)	0.0082 (7)	0.0062 (8)
O1	0.0445 (9)	0.0987 (14)	0.0438 (9)	−0.0153 (9)	−0.0083 (7)	0.0067 (9)
N1	0.0455 (10)	0.0500 (10)	0.0318 (9)	−0.0055 (8)	0.0045 (7)	0.0017 (7)
N2	0.0376 (9)	0.0480 (10)	0.0393 (10)	−0.0067 (8)	0.0023 (7)	−0.0029 (8)
C4	0.0358 (10)	0.0455 (11)	0.0307 (10)	0.0003 (9)	−0.0004 (8)	−0.0013 (8)
C3	0.0388 (10)	0.0384 (10)	0.0308 (10)	0.0031 (8)	0.0023 (8)	0.0000 (8)
C5	0.0650 (15)	0.0706 (17)	0.0544 (15)	−0.0116 (13)	0.0250 (12)	0.0023 (12)
C1	0.0653 (15)	0.0642 (15)	0.0299 (10)	−0.0058 (12)	−0.0055 (10)	0.0061 (10)
C2	0.0572 (14)	0.0649 (15)	0.0377 (12)	−0.0054 (12)	−0.0113 (10)	−0.0033 (10)
C6	0.0440 (13)	0.0738 (17)	0.0774 (19)	−0.0156 (12)	0.0118 (12)	−0.0092 (14)

### 1,4-Dimethylpiperazine-2,3-dione Geometric parameters (Å, º)

**Table:**

O2—C3	1.218 (2)	C5—H5B	0.9600
O1—C4	1.222 (2)	C5—H5C	0.9600
N1—C4	1.331 (3)	C1—C2	1.474 (3)
N1—C1	1.452 (3)	C1—H1A	0.9700
N1—C5	1.461 (3)	C1—H1B	0.9700
N2—C3	1.333 (3)	C2—H2A	0.9700
N2—C6	1.457 (3)	C2—H2B	0.9700
N2—C2	1.462 (3)	C6—H6A	0.9600
C4—C3	1.537 (3)	C6—H6B	0.9600
C5—H5A	0.9600	C6—H6C	0.9600
			
C4—N1—C1	121.47 (18)	N1—C1—C2	112.27 (18)
C4—N1—C5	120.23 (19)	N1—C1—H1A	109.2
C1—N1—C5	117.27 (18)	C2—C1—H1A	109.2
C3—N2—C6	119.7 (2)	N1—C1—H1B	109.2
C3—N2—C2	121.30 (18)	C2—C1—H1B	109.2
C6—N2—C2	118.05 (19)	H1A—C1—H1B	107.9
O1—C4—N1	124.1 (2)	N2—C2—C1	110.93 (19)
O1—C4—C3	118.12 (18)	N2—C2—H2A	109.5
N1—C4—C3	117.77 (17)	C1—C2—H2A	109.5
O2—C3—N2	123.9 (2)	N2—C2—H2B	109.5
O2—C3—C4	118.32 (18)	C1—C2—H2B	109.5
N2—C3—C4	117.82 (17)	H2A—C2—H2B	108.0
N1—C5—H5A	109.5	N2—C6—H6A	109.5
N1—C5—H5B	109.5	N2—C6—H6B	109.5
H5A—C5—H5B	109.5	H6A—C6—H6B	109.5
N1—C5—H5C	109.5	N2—C6—H6C	109.5
H5A—C5—H5C	109.5	H6A—C6—H6C	109.5
H5B—C5—H5C	109.5	H6B—C6—H6C	109.5
			
C1—N1—C4—O1	−175.3 (2)	N1—C4—C3—O2	−170.0 (2)
C5—N1—C4—O1	−7.2 (3)	O1—C4—C3—N2	−170.1 (2)
C1—N1—C4—C3	5.3 (3)	N1—C4—C3—N2	9.4 (3)
C5—N1—C4—C3	173.39 (19)	C4—N1—C1—C2	−35.3 (3)
C6—N2—C3—O2	−3.8 (3)	C5—N1—C1—C2	156.3 (2)
C2—N2—C3—O2	−172.4 (2)	C3—N2—C2—C1	−37.7 (3)
C6—N2—C3—C4	176.84 (19)	C6—N2—C2—C1	153.5 (2)
C2—N2—C3—C4	8.3 (3)	N1—C1—C2—N2	49.3 (3)
O1—C4—C3—O2	10.5 (3)		

### 1,4-Dimethylpiperazine-2,3-dione Hydrogen-bond geometry (Å, º)

**Table:**

*D*—H···*A*	*D*—H	H···*A*	*D*···*A*	*D*—H···*A*
C2—H2*A*···O2^i^	0.97	2.49	3.419 (3)	161
C5—H5*C*···O2^ii^	0.96	2.54	3.481 (3)	168

Symmetry codes: (i) *x*−1/2, −*y*+1/2, *z*−1/2; (ii) *x*+1/2, −*y*+1/2, *z*−1/2.

## References

[bb1] Agilent (2012). *CrysAlis PRO* and *CrysAlis RED*. Agilent Technologies Ltd, Yarnton, England.

[bb2] Bogatcheva, E., Hanrahan, C., Nikonenko, B., Samala, R., Chen, P., Gearhart, J., Barbosa, F., Einck, L., Nacy, C. A. & Protopopova, M. (2005). *J. Med. Chem.* **49**, 3045–3048. 10.1021/jm050948+ PMC4869334 16722620

[bb3] Brockunier, L. L., He, J., Colwell, L. F. Jr, Habulihaz, B., He, H., Leiting, B., Lyons, K. A., Marsilio, F., Patel, R. A., Teffera, Y., Wu, J. K., Thornberry, N. A., Weber, A. E. & Parmee, E. R. (2004). *Bioorg. Med. Chem. Lett.* **14**, 4763–4766. 10.1016/j.bmcl.2004.06.065 15324904

[bb4] Ge, Y., Han, Z., Wang, Z. & Ding, K. (2019). *J. Am. Chem. Soc.* **141**, 8981–8988. 10.1021/jacs.9b02920 31079460

[bb5] Gompper, R., Kellner, R. & Polborn, K. (1992). *Angew. Chem. Int. Ed. Engl.* **31**, 1202–1205.

[bb6] Groom, C. R., Bruno, I. J., Lightfoot, M. P. & Ward, S. C. (2016). *Acta Cryst.* B**72**, 171–179. 10.1107/S2052520616003954 PMC4822653 27048719

[bb7] Haraguchi, R., Takada, Y. & Matsubara, S. (2015). *Org. Biomol. Chem.* **13**, 241–247. 10.1039/c4ob01474j 25381867

[bb8] Krause, L., Herbst-Irmer, R., Sheldrick, G. M. & Stalke, D. (2015). *J. Appl. Cryst.* **48**, 3–10. 10.1107/S1600576714022985 PMC4453166 26089746

[bb9] Sheldrick, G. M. (2015*a*). *Acta Cryst.* A**71**, 3–8.

[bb10] Sheldrick, G. M. (2015*b*). *Acta Cryst.* C**71**, 3–8.

[bb11] Spek, A. L. (2020). *Acta Cryst.* E**76**, 1–11. 10.1107/S2056989019016244 PMC6944088 31921444

[bb12] Sterns, M., Patrick, J. M., Patrick, V. A. & White, A. H. (1989). *Aust. J. Chem.* **42**, 349.

